# Supplementary material for: Major sulfonate transporter Soa1 in Saccharomyces cerevisiae and considerable substrate diversity in its fungal family
Source: Nat Commun. 2017 Feb 6;8:14247. doi: 10.1038/ncomms14247 (PMC5303821; doi:10.1038/ncomms14247)
Supplement: Supplementary Information — Supplementary Figures. [file ncomms14247-s1.pdf]

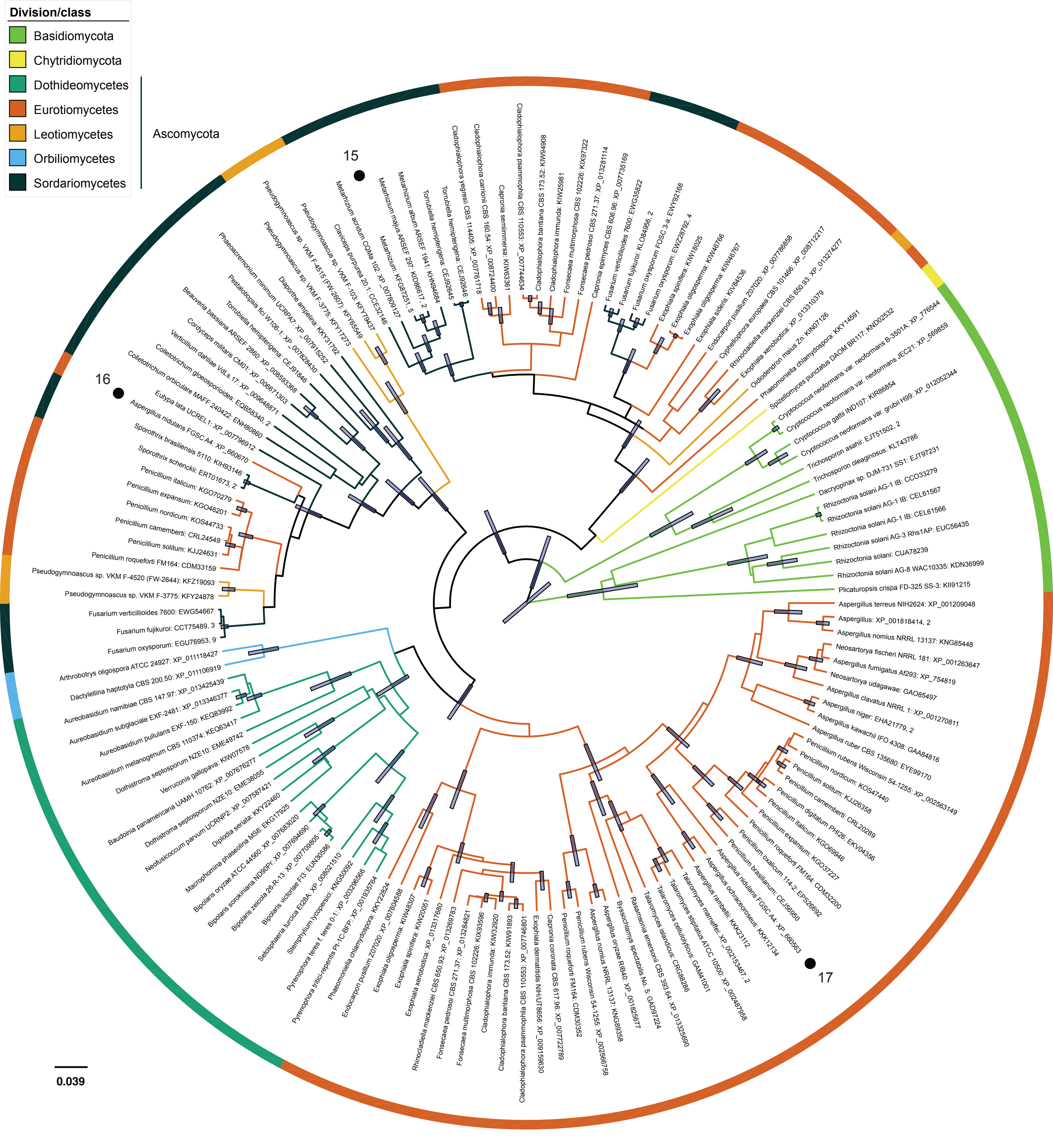

Supplementary Figure 1. Group 4 of the phylogenetic tree of alternative sulfate and sulfonate transporter orthologs. Details about the phylogenetic methods are described in the text. The scale bar shows units from the root of the collapsed node (0.385) to the tips (0).

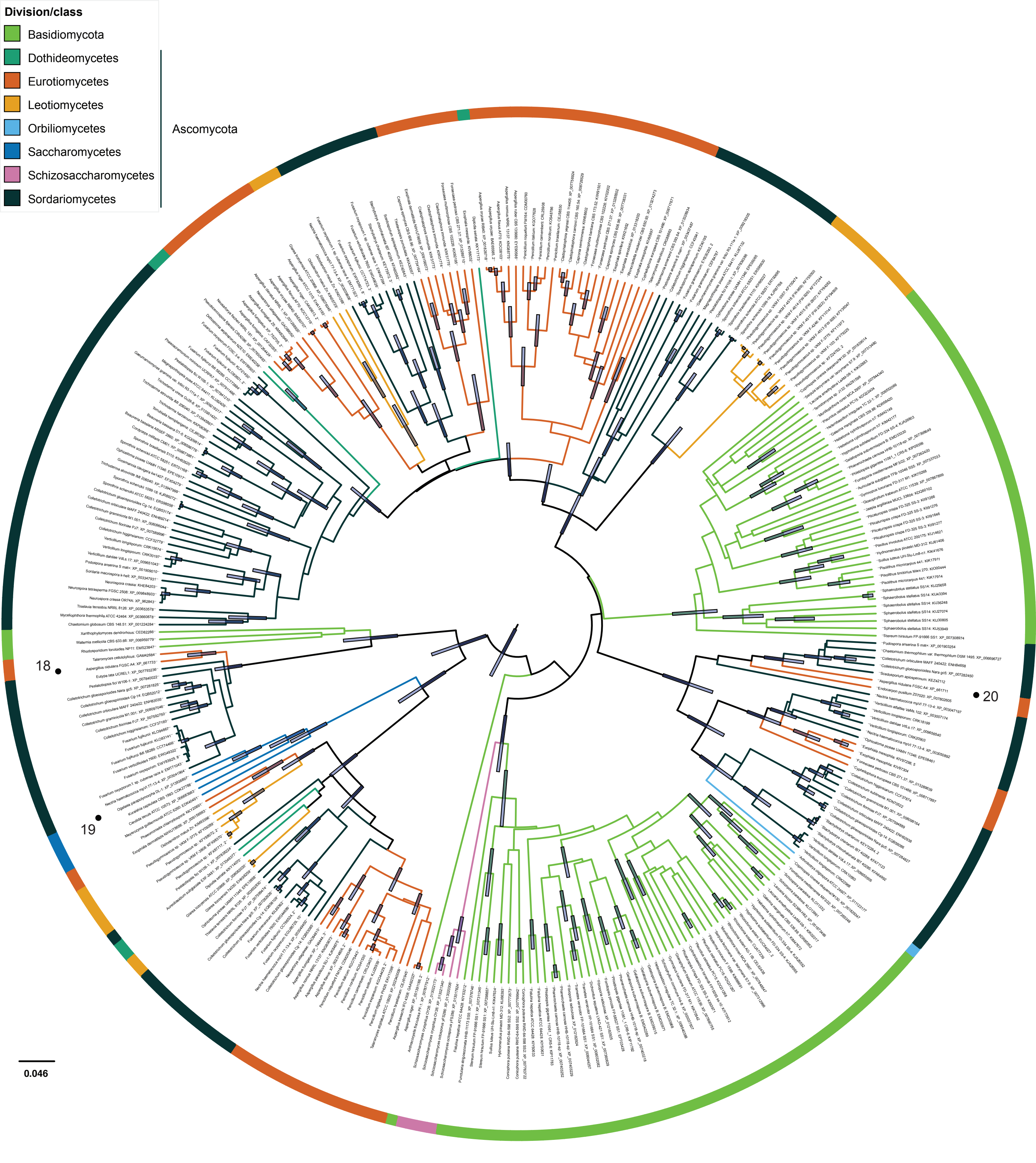

Supplementary Figure 2. Group 5 of the phylogenetic tree of alternative sulfate and sulfonate transporter orthologs. Details about the phylogenetic methods are described in the text. The scale bar shows units from the root of the collapsed node (0.423) to the tips (0).

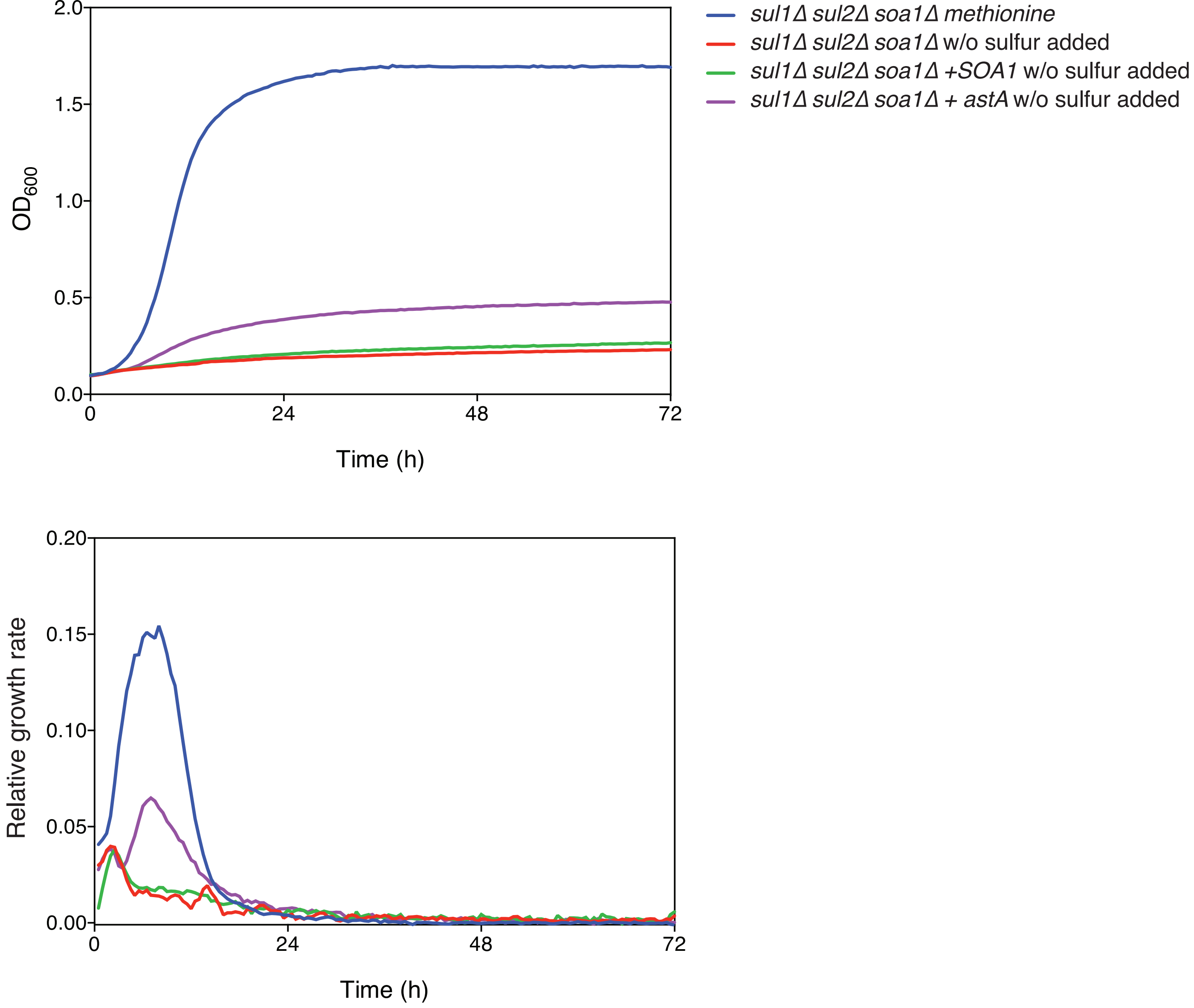

Supplementary Figure 3. Residual growth in sulfur-free medium without additional sulfur source added. Residual growth of the *sul1Δ sul2Δ soa1Δ* strain and the same strain with either *SOA1* or *ASTA* re-inserted into the *soa1Δ* locus in sulfur-free medium without any additional source of sulfur. Growth with methionine as positive control is only shown for the *sul1Δ sul2Δ soa1Δ* strain for simplicity. It is the same for the other strains. Relative growth rates $(X_t-X_{t-1})(X_{t-1})^{-1}$ were calculated and smoothed on 4 points with a second degree polynomial. The initial residual growth phase for the *sul1Δ sul2Δ soa1Δ* strain occurs in the first 2-2.5 h, whereas for the *sul1Δ sul2Δ soa1Δ + ASTA* strain a second residual growth phase occurs at a similar time as that of the maximal growth rate observed with methionine.
